# Supplementary material for: A Novel DUF569 Gene Is a Positive Regulator of the Drought Stress Response in Arabidopsis
Source: Int J Mol Sci. 2021 May 18;22(10):5316. doi: 10.3390/ijms22105316 (PMC8158135; doi:10.3390/ijms22105316)
Supplement: Supplementary file 1 [file ijms-22-05316-s001.zip › ijms-1200027-supplementary.pdf]

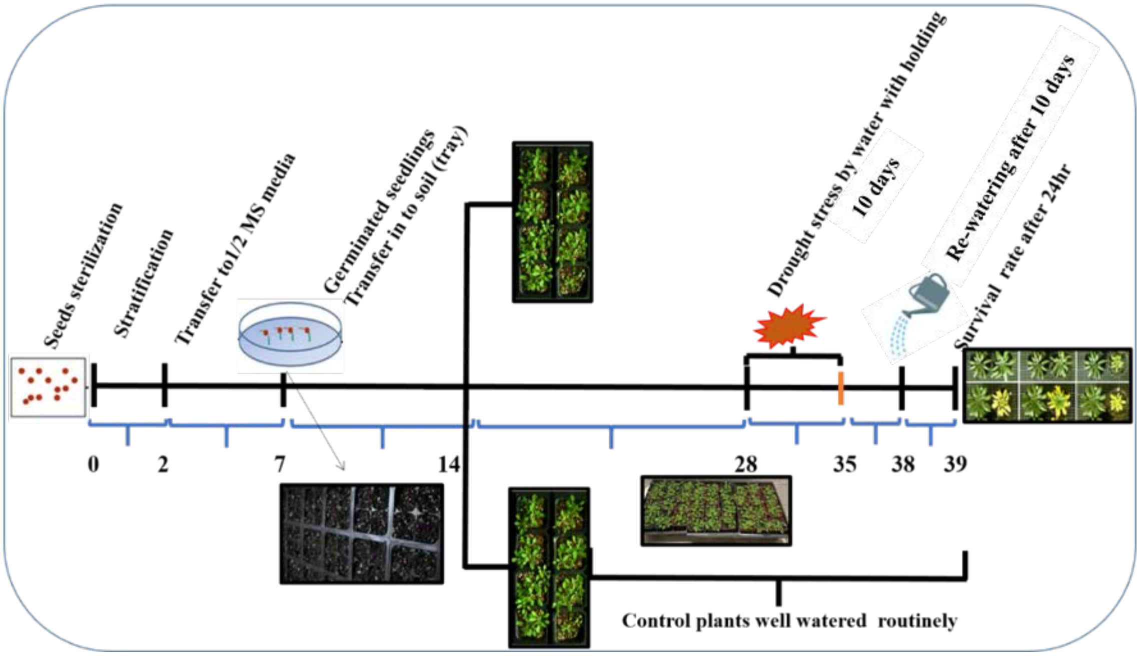

Figure S1 Illustration of drought assay through workflow scheme

Table S1 List of the primers used during (Drought) study

S.NO	Name	Forward primer (5´-3´)	Reverse primer (5´-3´)
1	Actin	GCTGGACGTGACCTTACTGA	CCATCTCCTGCTCGTAGTCA
2	*AtAPX1*	GTCCATTCGGAACAATGAGGTTTGAC	GTGGGCACCAGATAAAGCGACAAT
3	*AtABI2*	GTTCTTGTTCTGGCGACGGAGC	CCATTAGTGACTCGACCATCAAG
4	*AtABA2*	ACGGTTGATGATGTAGCGAACGCTGTT	CATCTGAAGACTTTAAAGGAGTGGTTAG
5	*AtABA3*	CGTCGTCAGTGGAAGGTTTC	AATTTCACCGGTCAGACCCT
6	*AtDREB1A*	ACGAGTCTTCGGTTCCTCA	ACAAACCCACTTACCGGAGT
7	*AtDREB2A*	GACCTAAATGGCGACGATGT	TCGAGCTGAAACGGAGGTAT
8	*ATMAX2*	CCGAGCCAGAGTTTGGGTTA	GTGCGAAACCGATTGTGTCC
9	*ATRGA1*	TTGTCCAACCACGGGACTTC	AGCTCGTCGTCCATGTTACC
10	*ATHK3*	GGGCATCTCTTCTGGATGCT	GATCACCGACAGACCAACT
11	*NIA1*	AGTACGGTAAATTCTGGTGCTGGTG	CCCTATCTCTCCTCTATGAGGCTTG
12	*NIA2*	GACGCCGAACTCGCCGACGAAG	TGTCTCTCCACCATCTACCGTGACCTC
13	*GSNOR1*	CGTCGCCAAAATTGATCCTACTGC	TCACTTCCTGAATTGGCTTGTCGT
14	*AtNCED3*	TCCTCTGTTTCGTTCACGACG	CGTACGGAACCCTTGACGGA

Table S2. Regulation of amino acids (μg/g^{-1} DW) in Arabidopsis genotypes under drought stress.

Treatment	Asp	Thr	Met	ILE	Ser	Glu	Leu	Tyr	Gly	Phe	Lys	Cys	Val	His	Arg	Ala	Pro
Col-0 (WC)	19.56 ± 0.49	4.73 ± 0.5	0.18 ± 0.11	11.46 ± 0.43	5.59 ± 0.48	26.46 ± 0.41	22.58 ± 0.41	0.51 ± 0.05	12.57 ± 0.16	8.77 ± 0.39	17.81 ± 0.44	0.65 ± 0.07	14.84 ± 0.51	7.03 ± 0.43	10.61 ± 0.30	13.47 ± 0.4	17.16 ± 0.58
atduf569 (WC)	22.07 ± 0.61	6.11 ± 0.27	0.26 ± 0.06	13.34 ± 0.49	6.91 ± 0.4	31.46 ± 0.76	26.08 ± 0.88	0.56 ± 0.13	14.38 ± 0.42	9.31 ± 0.505	19.95 ± 0.26	0.76 ± 0.13	16.03 ± 0.49	8.39 ± 0.58	12.18 ± 0.32	15.0 ± 0.54	20.79 ± 0.56
Col-0 (Drought)	23.38 ± 0.52	9.99 ± 0.72	0.15 ± 0.08	13.33 ± 0.52	8.82 ± 0.35	31.01 ± 1.09	23.72 ± 0.60	0 ± 0	14.46 ± 0.67	13.54 ± 0.32	19.05 ± 0.45	1.07 ± 0.08	15.69 ± 0.64	6.68 ± 0.46	12.35 ± 0.52	13.69 ± 0.62	20.85 ± 0.71
atduf569 (Drought)	21.36 ± 0.45	9.22 ± 0.92	0.23 ± 0.079	13.44 ± 0.79	8.27 ± 0.48	28.47 ± 0.49	24.89 ± 1.24	0 ± 0	15.78 ± 0.99	12.33 ± 0.61	20.29 ± 0.87	1.09 ± 0.01	16.09 ± 0.57	7.34 ± 0.49	10.91 ± 0.59	13.88 ± 0.85	18.8 ± 0.71

Notes: WC, water control; amino acids represented as Asp, aspartic acid; Thr, threonine; Met, methionine; ILE, isoleucine; Ser, serine; Glu, glutamic acid; Leu, leucine; Tyr, tyrosine; Gly, glycine; Phe, phenylalanine; Lys, lysine; Cys, cysteine; Val, valine; His, histidine; Arg, arginine; Ala, alanine; Pro, proline. Data are means (±SD) of at least three replications.
